# Synthesis of Heart/Dumbbell-Like CuO Functional Nanostructures for the Development of Uric Acid Biosensor

**DOI:** 10.3390/ma11081378

**Published:** 2018-08-08

**Authors:** Zafar Hussain Ibupoto, Aneela Tahira, Hamid Raza, Gulzar Ali, Aftab Ahmed Khand, Nabila Shah Jilani, Arfana Begum Mallah, Cong Yu, Magnus Willander

**Affiliations:** 1 State Key Laboratory of Electroanalytical Chemistry, Changchun Institute of Applied Chemistry, Chinese Academy of Sciences, Renmin St. 5625, Changchun 130022, China; congyu@ciac.jl.cn; 2 Institute of Chemistry, University of Sindh, 76080 Jamshoro, Pakistan; gulzaralichemist@gmail.com (G.A.); arfana30@gmail.com (A.B.M.); 3 Department of Science and Technology, Campus Norrköping, Linköping University, SE-60174 Norrköping, Sweden; aneela.tahira@liu.se; 4 Department of Anaesthesia Liaquat, University of Medial and Health Sciences, 76080 Jamshoro, Pakistan; hamid.raza@gmail.com; 5 School of Life Sciences, Tsinghua University, Beijing 100084, China; aftab_k97@yahoo.com; 6 Institute of Plant Sciences, University of Sindh, 76080 Jamshoro, Pakistan; nabila_jilani@hotmail.com; 7 Department of Science and Technology, Campus Norrköping, Linköping University, SE-60174 Norrköping, Sweden; magnus.willander@liu.se

**Keywords:** CuO nanostructures, vitamin B_12_, uric acid biosensor, potentiometric response

## Abstract

It is always demanded to prepare a nanostructured material with prominent functional properties for the development of a new generation of devices. This study is focused on the synthesis of heart/dumbbell-like CuO nanostructures using a low-temperature aqueous chemical growth method with vitamin B_12_ as a soft template and growth directing agent. CuO nanostructures are characterized by scanning electron microscopy (SEM), X-ray diffraction (XRD), and X-ray photoelectron spectroscopy (XPS) techniques. CuO nanostructures are heart/dumbbell like in shape, exhibit high crystalline quality as demonstrated by XRD, and have no impurity as confirmed by XPS. Apparently, CuO material seems to be porous in structure, which can easily carry large amount of enzyme molecules, thus enhanced performance is shown for the determination of uric acid. The working linear range of the biosensor is 0.001 mM to 10 mM with a detection limit of 0.0005 mM and a sensitivity of 61.88 mV/decade. The presented uric acid biosensor is highly stable, repeatable, and reproducible. The analytical practicality of the proposed uric acid biosensor is also monitored. The fabrication methodology is inexpensive, simple, and scalable, which ensures the capitalization of the developed uric acid biosensor for commercialization. Also, CuO material can be used for various applications such as solar cells, lithium ion batteries, and supercapacitors.

## 1. Introduction

Cupric oxide (CuO) is a member of first row transition metal oxides with unique properties and advantages such as its inexpensive nature and abundance on Earth [1,2]. CuO, with its controlled shape and dimension, has received more attention due to its potential applications in various fields such as catalysis [3,4,5,6,7,8], batteries [9,10,11,12], solar cells [13,14], supercapacitors [15,16], sensors [17,18,19], and photodetectors [20,21]. Besides this, CuO as nanostructured materials can reveal size dependent physical and chemical properties, along with high surface area and quantum confinement [22]. Active research activities have been carried out for the synthesis of nanostructured CuO materials with well-defined morphology and size [2]. Thus, numerous morphologies of CuO are produced including nanoparticles, nanoneedles, nanowhiskers, nanowires, nanoshuttles, nanorods, nanotubes, nanoleaves, and nanoribbons via wet chemistry and physical methods [23,24,25,26,27,28,29,30,31]. Moreover, the complex nanostructures of CuO are also synthesized including nanoellipsoids [32], peanut-like nanostructures [33], nano-dendrites [34], prickly/layered microspheres [35], and dandelion-like hollow morphology [36]. The wet chemical method has more importance over other existing methods due to its low cost, simplicity, and gives a high yield of nanostructured material. Due to fascinating electrochemical properties of CuO, it is able to be a main component of electrochemical sensors, especially potentiometric sensors [37].

Uric acid (UA) is the major product of purine metabolism and its release in urine is because of purines that are formed in the catabolism of the dietary and endogenous nucleic acid. The formation of uric acid in excess can result in precipitation in the kidney and it hinders urine excretion. The possibility of gout may be observed due to the metabolism of uric acid [38]. Several studies have shown that the formation of excessive uric acid in human blood is highly risky and can cause cardiovascular diseases [39]. Therefore, the estimation of uric acid in human physiological fluids is very important for the early diagnosis of patients who are victim to a wide range of abnormalities due to variations in purine metabolism. Currently, several uric acid biosensors are reported from different research groups [40,41,42,43,44]. Many of these biosensors are based on an amperometry technique [45,46,47,48]. These biosensors suffer severe disadvantages that hinder their practical applications due to their working potential at 0.7 V [49]. The relatively high electrode potential makes other competing species oxidize on the surface of electrode [50]. This kind of limitation and interference can be avoided by using the potentiometric configuration that works at a negligible bias voltage as previously reported in the several studies [51,52,53,54]. The vitamin B_12_ has been used as a reducing and capping agent for the preparation of noble metal nanoparticles [55].

The template assisted nanostructured materials have the advantage of fast growth nucleation and in getting controlled morphology of nanomaterials. The vitamin B_12_ has biocompatibility with metal oxide nanostructures, which is being presented in this work as an evident for the growth of other metal oxides. It is for the first time that vitamin B_12_ is used as a growth directing agent for tuning the morphology of CuO nanostructures. The present study is focused on the preparation of heart/dumbbell-like CuO nanostructures using vitamin B_12_ with excellent functional properties during the development of a potentiometric uric acid biosensor for the first time.

In this research work, vitamin B_12_ is used as growth directing agent to control the morphology of CuO nanostructures using a low-temperature aqueous chemical growth method. The CuO nanostructured material is characterized by SEM, XRD, and XPS techniques. The functional properties of nanostructured CuO are demonstrated in the development of a sensitive, selective, stable, reproducible, and repeatable uric acid biosensor. The proposed potentiometric configuration was selectivity used in the determination of uric acid from the real samples, which confirms the practicality of the presented analytical device.

## 2. Experimental Section

### 2.1. Chemicals Used

Copper nitrate pentahydrate (Cu (NO_3_)_2_·5H_2_O, 25% ammonia, vitamin B_12_, uricase (E.C. 1.7.3.3), 25 units/1.5 mg from Arthrobacter globiformis, uric acid, d-glucose, ascorbic acid, glutaraldehyde, hydrochloric acid (HCl), sodium hydroxide (NaOH), sodium chloride (NaCl), potassium chloride (KCl), disodium hydrogen phosphate (Na_2_HPO_4_), and potasium dihydrogen phosphate (KH_2_PO_4_) were purchased from Sigma Aldrich, Jilin, china. A phosphate buffer solution of 10 mM was made by mixing appropriate quantities of NaCl, KCl, Na_2_HPO_4_, and KH_2_PO_4_ in deionized water and a fixed pH of 7.4 for the phosphate buffer solution was obtained by adding a certain volume of 1 M NaOH, and 1 M HCl. A fresh uric acid solution was prepared in the phosphate buffer solution and kept at 4 °C. The low concentration solutions were prepared using a dilution method. All the chemicals used were of analytical grade and used without further any purification.

### 2.2. Synthesis of CuO Nanostructures Using a Low-Temperature Aqueous Chemical Growth Method with Vitamin B_12_ on Gold Coated Glass Substrates

To modify the surface of the gold-coated glass substrates with CuO nanostructures, a two-step methodology was followed. First, glass substrates were cleaned with acetone and deionized water in an ultrasonic bath, then dried with flowing nitrogen gas. Afterwards, the glass substrates were fixed inside the vacuum chamber of a Satis, Norrköping, Sweden (CR 725) evaporator. A thin layer of 10 nm of chromium was deposited on the glass substrates as an adhesive layer, followed by the 100 nm thickness deposition of the gold layer. In the second step, gold-coated glass substrates were again sonicated in acetone in an ultrasonic bath for 15 min and dried using flowing nitrogen gas, then a seed layer of CuO nanoparticles was spin-coated using a spin coater at 2500 rpm for 30 s and seed coated substrates were annealed at 120 °C for 30 min in order to get a firm binding of seed particles on the substrates. Afterwards, a 25 mM copper nitrate pentahydrate solution was prepared in 100 mL deionized water and 5 mL of 25% ammonia was added in the solution. In order to facilitate the growth process and to get CuO nanostructured material of desired properties, 0.5 g of vitamin B_12_ was used as a soft template. Then, the seed-coated gold-coated glass substrates were fixed in a Teflon sample holder and kept in the growth solution. The beaker was tightly sealed using aluminum foil and kept at 95 °C for 24 h. Afterwards, the CuO-modified substrates were collected and washed with the deionized water in order to remove the residual particles from the surface of the nanostructured CuO and dried with flowing nitrogen gas.

### 2.3. Material Characterization

The morphology and structural investigations of nanostructured CuO were performed by SEM at a 15 kV accelerating voltage. The crystal structure was studied using X-ray powder diffraction (XRD) with a Phillips (PW 1729, Tokyo, Japan) powder diffractometer associated with CuKα radiation (λ = 1.5418 Å at a generator voltage of 40 kV and a current of 40 mA). The XPS experiments were done using an ESC (A200, Sweden) spectrometer in highly vacuum of pressure of 10^−10^ mbar. The measurement chamber was equipped with a monochromatic Al (Kα) X-ray source employing photons of frequency (hν = 1486.6 eV).

### 2.4. The Immobilization of Uricase Enzymes on the Nanostructured CuO and Potentiometric Measurement

To immobilize uricase enzymes on nanostructured CuO, first uricase solution was prepared in a phosphate buffer solution of pH 7.4 using (3 mg/mL uricase) and 100 μL of glutaraldehyde was used as a cross-linker to avoid the self-enzyme reaction. Then, the CuO material was dipped in the enzyme solution for 5 min and the immobilized electrodes were dried in air at room temperature for 1 h. After the immobilization, the electrodes were kept at 4 °C when not in use. The potentiometric measurements for the sensing of uric acid were done against Ag/AgCl as a reference electrode using a Metrohm pH meter (Model 744, Beijing, China) by employing the uricase-immobilized nanostructured CuO material as a working electrode. All the experiments were performed at room temperature and all solutions were prepared in a 10 mM phosphate buffer solution of pH 7.4. The biosensor can be reused after rinsing with the buffer solution.

## 3. Results and Discussion

### 3.1. The Morphological, Structural, and Composition Studies of as Prepared CuO Nanostructures Using Vitamin B_12_

**Figure 1:**
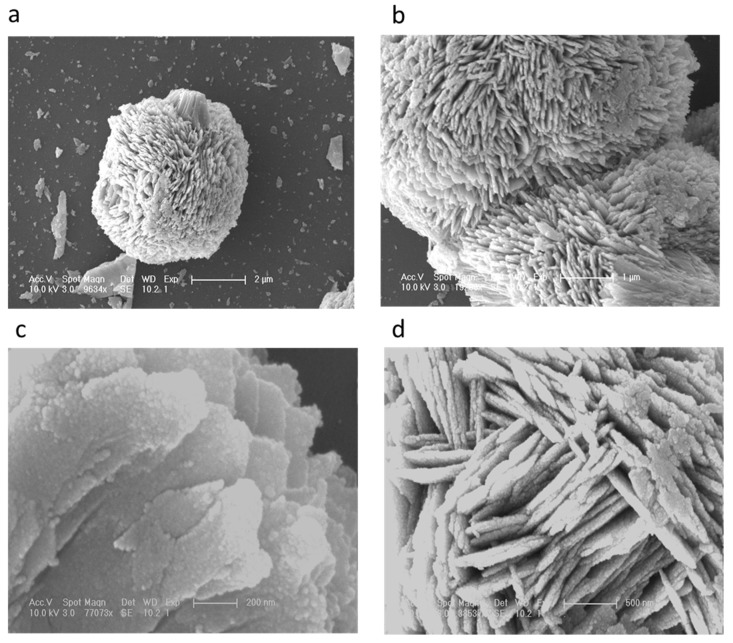
(**a**–**d**): SEM images of the vitamin B_12_ assisted synthesis of CuO nanostructures at different magnifications.

The distinctive SEM images at different magnifications for the nanostructured CuO material are shown in Figure 1a–d. It can be seen from the low magnification image Figure 1a that the morphology of CuO nanomaterial was heart/dumbbell-like, which is further verified at higher magnification and it exhibits a porous structure as depicted in Figure 1b. Furthermore, it can be seen that the formation of the heart/dumbbell-like morphology was comprised of thin sheets consisting of the nanoparticles on the surface as shown in Figure 1c. Figure 1d shows that the sheets in the structured CuO nanomaterial were well-packed with a thickness of 100 to 200 nm. The heart/dumbbell-like morphology was well controlled using vitamin B_12_ as the soft template that ensured the uniform growth of the CuO nanomaterial. Chemically, vitamin B_12_ contains several polar functional groups in its structure along with a small portion of non-polar cyclic structures, but the dominancy in the growth of the CuO heart/dumbbell-like nanostructure is governed by the polar group that allowed for the control of the kinetic process during the growth in water and resulted a unique morphology of the CuO nanomaterial. However, the carbon chains of vitamin B_12_ were acting as a soft template for the maturing heart/dumbbell structure. The actual role of vitamin B_12_ in the evolution of CuO nanostructures is still unclear, thus we are only providing a possible role of vitamin B_12_ based on the preliminary results.

**Figure 2:**
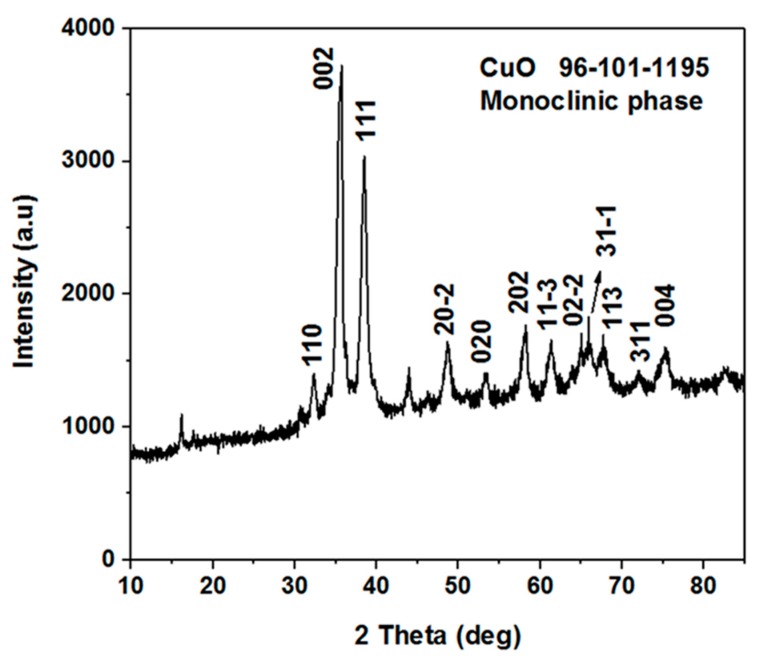
The XRD spectrum of the vitamin B_12_-assisted CuO nanomaterial.

Figure 2 shows the XRD patterns of the nanostructured CuO material prepared using a low-temperature aqueous chemical growth method. The measured diffraction patterns are according to the standard (JCPDS card no: 96-101-1195). The CuO nanomaterial exhibited the monoclinic phase and no other peak was detected, which confirms the high purity of the CuO nanomaterial.

**Figure 3:**
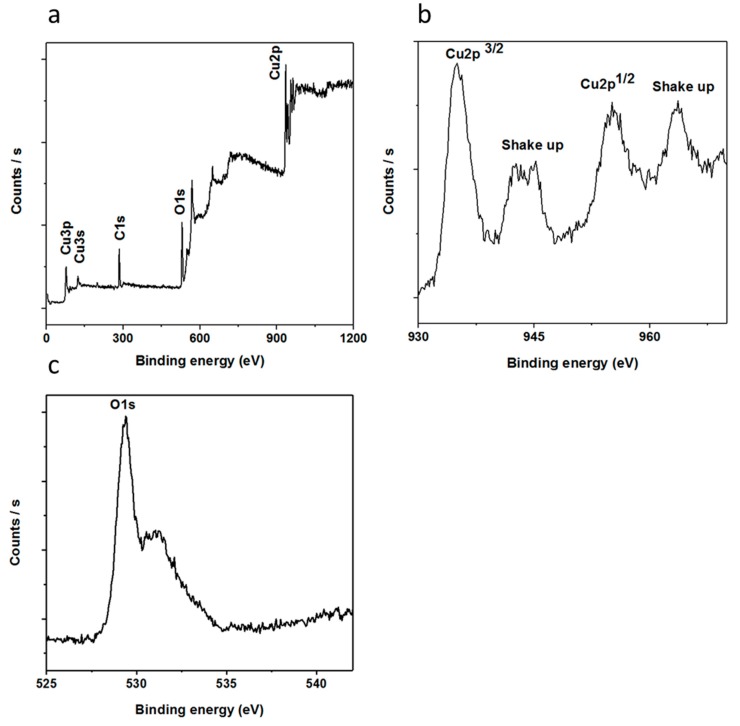
The XPS spectra of vitamin B_12_-assisted CuO nanostructures, (**a**) wide scan survey of elements present in the sample; (**b**) Cu 2p; (**c**) O 1s.

The chemical composition of the nanostructured CuO was further investigated using XPS measurements and the obtained spectra are shown in Figure 3. Figure 3a shows the wide scan survey for the elemental composition that has two distinctive peaks, where those at 284.00 and 531.00 eV represent C 1s and O 1s, respectively [56]. Additionally, the recorded peaks at 933.30, 121.10 and 77.00 eV could be attributed to Cu 2p, Cu 3s and Cu 3p respectively [57].

Figure 3b,c discloses the XPS spectra of Cu 2p and O 1s, respectively. In the case of Cu 2p, the recorded peak at 933.60 eV was assigned to the binding energy of Cu 2p3/2, which is in good agreement with the published work [58], as shown in Figure 3b. Moreover, two shake-up peaks show the clear evidence for the synthesis of a CuO compound using a low-temperature aqueous chemical growth method. Figure 3c shows the XPS spectrum of O 1s in which two peaks are observed that could be indexed to the O^2−^ in CuO at 529.47 eV and the peak at 531.15 eV is from the adsorbed oxygen, respectively. From composition point of view, XRD and XPS studies are in good agreement with each other.

### 3.2. The Potentiometric Response of the Proposed Uric Acid Biosensor Based on Uricase Immobilized Heart/Dumbbell-Like CuO Nanostructures

The potentiometric measurement was carried out via a two-electrode cell system using heart/dumbbell-like CuO nanostructures as an excellent transducer for the immobilization of uricase acting as a working electrode and silver-silver chloride (Ag/AgCl) as a reference electrode. All the experiments were performed at room temperature except for the temperature study. The output potential signal was measured in the phosphate buffer solution; thus, different concentrations of uric acid were prepared in the phosphate buffer solution of pH 7.4. The uricase immobilized heart/dumbbell-like CuO nanostructures were tested in different concentrations of uric acid ranging from 0.001 mM to 10 mM and the response of the biosensor was found according to Nernst’s equation:

E = E_0_ − 0.05916 V/n log [Reduced]/[Oxidized] (1)

**Figure 4:**
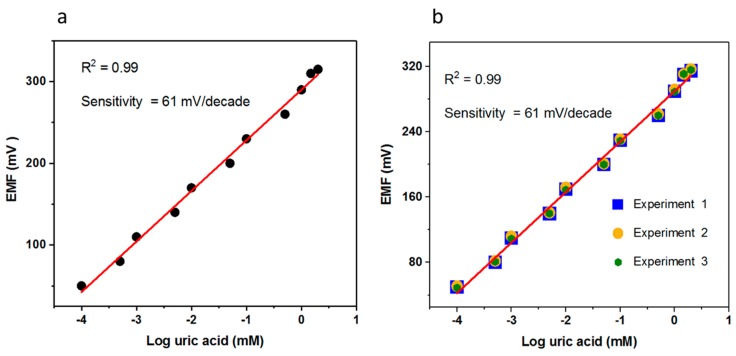
(**a**) The calibration fitting of the potentiometric uric acid biosensor for a concentration range of 0.0001–10 mM uric acid concentration; (**b**) the repeatability response of the uric acid biosensor in the concentration range of 0.0001–10 mM uric acid.

The sensing mechanics of the electrochemical uric acid biosensors was demonstrated by uricase. It was uricase that oxidized the uric acid into allantoin along with the formation of carbon dioxide and hydrogen peroxide. As the biosensor was working in the water, the allantoin most probably snatched a proton from the water and consequently there was the formation of an allantoinium ion that further interacted with the CuO nanostructures and at the surface of the CuO there was an accumulation of charges that were responsible for the output potential and was easily measurable by the potentiometric technique. The scale of the potential varied with respect to different concentrations of uric acid. The fabricated uric acid biosensor responded to a wide linear range of uric acid and the output potential was linearly related to the logarithmic concentrations of the uric acid, having a sensitivity of 61.88 mV/decade, which is very close to the Nernstian slope and it indicates that the proposed configuration of sensor system was in good agreement with the Nernstian response with a regression coefficient of 0.99. The limit of detection of the fabricated uric acid biosensor was found to be 0.0005 mM, which is estimated from the linear range as shown in Figure 4a. The repeatability was another way to notice the response of the same-immobilized CuO nanostructures electrode and its capability of reuse after rinsed with phosphate buffer solution and this experiment was repeated for the same electrode for three consecutive days. When the biosensor was not in use, it was kept at 4 °C, and this study has shown that the biosensor has the ability to survive for a long time by maintaining the sensitivity and linear range.

**Figure 5:**
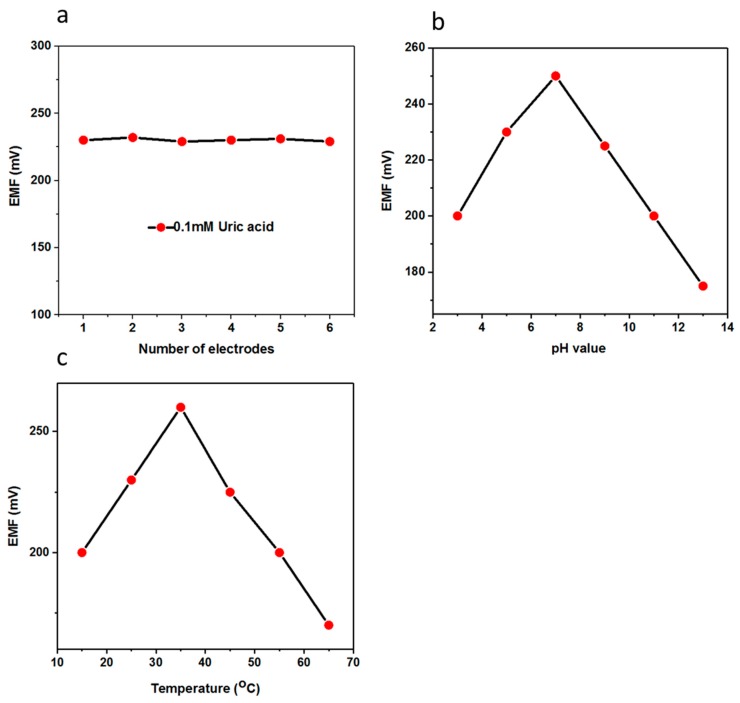
(**a**) The inter-electrode reproducibility of the uric acid biosensor in 0.1 mM uric acid; (**b**) the effect of pH on the output potential of the uric acid biosensor in 0.5 mM uric acid; (**c**) the effect of temperature on the potentiometric response of the uric acid biosensor in 0.5 mM uric acid.

For the performance evaluation of the biosensor, several parameters were investigated such as reproducibility, repeatability, selectivity, and stability. The reproducibility is an essential parameter for the performance evaluation of a fabricated biosensor. In order to ensure reliable reproducibility, six independent electrodes were fabricated by following same conditions as discussed above and the electrodes were immersed in 0.1 mM uric acid solution and the recorded response is shown in Figure 5a, which clearly indicates the excellent inter-electrode response of the fabricated biosensor using functional nanostructured CuO. The relative standard deviation of the electrode-to-electrode response was found to be less than 5%, which shows the promising analytical features of the proposed uric acid biosensor. The presented uric acid biosensor based on CuO nanostructures offers a decent hosting platform for the immobilization of uricase enzyme and ultimately demonstrates the sensitive and selective response to uric acid under physiological conditions. Owing to the porous structure of the CuO material, the uricase molecules were allowed to penetrate within the body of the dumbbell-like nanostructures. The uricase was firmly bound to the CuO surface to expose the highly attractive features for the oxidation of uric acid. The stability of the fabricated uric acid biosensor was evaluated for four weeks and the biosensor had the ability to maintain the linear range, detection limit, and sensitivity as shown in Table 1. The biosensor could be used for more than four weeks if the storage conditions were well-controlled. Importantly, the CuO nanostructures-based uric acid biosensor could easily be capitalized for the monitoring of uric acid from real samples as all experiments were performed in the same physiological conditions. Therefore, a recovery method was used for the real sample analysis using the presented uric acid biosensor as the driving candidate for the sensing of uric acid with a satisfactory performance as shown in Table 2.

The selectivity is the backbone of the fabricated biosensor because it is directly identifying the sensing element in the presence of other competing species. Several methods are used to monitor the selectivity of the potentiometric biosensors such as separate solution method, mixed solution method, matched potential method, and unbiased selective coefficients. In the current study, a separate solution method was used to measure selectivity coefficients according to our reported work [59] and the obtained values are fairly constant as given in Table 3. In the human blood, the common interfering species are ascorbic acid, urea, and glucose during the sensing of uric acid. Using 1 mM uric acid and 0.1 mM of each of the interfering species, selectivity coefficients were calculated, which are fairly constant. This study has strengthened the claim that the uric acid biosensor is highly selective for the quantification of uric acid in physiological conditions.

### 3.3. pH and Temperature Studies

The aim of this study was to find the suitable pH conditions under which the biosensors based on metal oxides nanostructures seemed to be stable because many of the metal oxides do not have a good figure of merit in harsh conditions, and also to ensure the physiological pH for which the biosensor needed to be in the driving position. pH has a direct influence on the activity of enzymes and the change of charges at the surface of the electrode strongly effects the output potential of the sensor. The pH-based potentiometric signal was recorded in the 0.5 mM uric acid solution and the biosensor showed the optimum response close to pH 7, which is in good agreement with the performance of uricase enzyme; however, for higher pH values, the response of the biosensor degraded, which could be due to the loss of enzymatic activity and also the slow dissolution of metal oxides in the analyte solution as shown Figure 5b. Thus, pH 7.4 was chosen for all the measurements except the pH study.

Under the controlled experimental setup, the effect of different temperatures on the performance of the biosensor was examined in a 0.1 mM uric acid solution in the temperature range of 15 to 65 °C as shown in Figure 5c. It can be seen that there was a gradual increase of output potential when the temperature was increased, and at 35 °C the maximum response was recorded, which indicates that the kinetics of enzymatic activity was enhanced, and at high temperature the enzyme starts to denature and loose its activity, and thus the output signal dropped. Keeping in mind the ease of the experimental setup to avoid the solution evaporation, the measurements were therefore performed at room temperature.

The analytical results of the presented biosensor are compared with the existing uric acid biosensors in terms of linear range and sensitivity as given in Table 4. It is obvious that the proposed uric biosensor exhibited a wide linear range due to the porous nature of CuO nanostructures that carried large amounts of uricase during immobilization and provided a large surface for uric acid molecules to undergo easy oxidation.

## 4. Conclusions

In this study, heart/dumbbell-like CuO nanostructured material was synthesized uisng vitamin B_12_ as a growth-directing agent and template for the facilitation of the growth process using a low-temperature aqueous chemical growth method. CuO nanostructures were investigated uisng SEM, XPS, and XRD techniques. Using these nanostructures of CuO, uricase enzyme was immobilized on them and used for the development of a stable, sensitive, selective, reproducible, and repeatable uric acid biosensor. The nanostructures of CuO are porous, which allowed the uricase molecules to reside within those pores to further allow the fast oxidation of uric acid, and finally we have a successful and an alternative analytical device for the monitoring of uric acid. The obtained results were unique and can be capitalized to commercialize the uric acid biosensor because the fabrication process is simple, cost effective, and scalable. We propose the functional properties of the prepared nanostructured CuO material in the field of lithium ion batteries, solar cells, and supercapacitors based on its functional properties.

## Figures and Tables

**Table 1:** The storage stability proposed uric acid biosensor based on CuO nanostructures.

No of Weeks	Linear Range Uric Acid (mM)	Sensitivity (mV/Decade)	Limit of Detection Uric Acid (mM)
1	0.001–10	61.88	0.0005
2	0.002–10	61	0.0005
3	0.001–10	61.58	0.0004
4	0.0025–10	60	0.0003

**Table 2:** The recovery method results for the analytical reliability of uric acid biosensor.

Spiked Concentration of Uric Acid (Mm)	Uric Acid Conc. as Quantified by Proposed Biosensor	% Uric Acid Biosensor Recovery
0	1.5	-
1.8	2	111.11
3.5	3.7	105.71
2.5	2.4	96
4.1	4.2	102.43

**Table 3:** The calculated selectivity coefficients for the interfering species using separate solution method using 0.1 mM solution of each interfering substance.

Interfering Species (B)	Log K^pot^_uric acid_, B
Ascorbic acid	2.5
Urea	1.9
Glucose	2.25

**Table 4:** The comparison of presented uric acid biosensor with existing biosensors.

Electrode Material	Technique	Linear Range mM	Sensitivity mV/Decade	References
ZnO nanowires	Potentiometry	0.001–1	29	[60]
ZnO nanotubes	Potentiometry	0.05–1.5	68	[61]
ZnO nanoflakes	Potentiometry	0.0005–1.5	66	[62]
ZnO nanorods	Amperometry	0.005–1	----------	[63]
ZnO nanoparticles	Amperometry	0.00–1	----------	[64]
PEDOT/Palladium	Differential pulse voltammetry	0.007–0.011	----------	[65]
PrGO	Differential pulse voltammetry	0.3	----------	[66]
Graphene-poly(acridine red)/GCE	Differential pulse voltammetry	0.008–0.15	----------	[67]
RGO–AuNPs–CSHMs	Differential pulse voltammetry	0.001–0.3	----------	[68]
CuO nanostrucutures	Potentiometery	0.001–10	61	This work
